# Biological Activity of Hybrid Molecules Based on Major Constituents of *Cinnammomun verum* and *Thymus vulgaris* Essential Oils

**DOI:** 10.3390/life13020499

**Published:** 2023-02-11

**Authors:** Juan Valverde Sancho, Cristina Carreño Amate, María del Mar Caparrós Pérez, Omar Santana Méridas, Luis F. Julio

**Affiliations:** 1 Green Chemistry Area, R&D Department, Kimitec Group, Paraje Cerro de Los Lobos, 04738 Vícar, Spain; 2 Phytopathology Area, R&D Department, Kimitec Group, Paraje Cerro de Los Lobos, 04738 Vícar, Spain; 3 Phytochemistry Area, R&D Department, Kimitec Group, Paraje Cerro de Los Lobos, 04738 Vícar, Spain

**Keywords:** *Cinnamomun verum*, *Thymus vulgaris*, essential oils, hybrid molecules, antifungal, lead discovery, enzymatic catalysis, cytotoxicity

## Abstract

Plants have been used by humans since ancient times due their antimicrobial and medicinal properties. Essential oils (EOs) are complex mixtures of secondary plant metabolites, including terpenoids, phenylpropanoids, and other aromatic compounds. *Cinnamomun verum* and *Thyme vulgaris* EOs and their organic extracts exert numerous biological activities because of their major compounds, particularly thymol, carvacrol, eugenol, and benzoic and cinnamic acid. The structural motifs presented by these phytochemicals are responsible for their biological activities. Modification or hybridization of these structures could lead to new bio-based compounds with improved efficacy or multiple modes of action. In this work, we aimed to develop reliable methods of obtaining six hybrid molecules from the major constituents of *C. verum* and *T. vulgaris* EOs. For the first time, we tested their efficacy in the inhibition of the mycelium growth and spore germination of two of the most important phytopathogenic fungi, *Fusarium oxysporum* and *Colletotrichum gloeosporioides*, and one opportunistic human pathogen, *Aspergillus niger*. The cytotoxic activity of the obtained hybrids was assessed using the brine shrimp lethality assay. In addition, we report for the first time a biocatalytic process for the obtention of these bioactive hybrid molecules. The results of this work enable the possibility of using hybrid molecules based on the major constituents of EOs as active ingredients in strategic industries such as agriculture, aquaculture, and pharmaceuticals.

## 1. Introduction

Plants are one of the richest and most versatile chemical factories on the planet for obtaining natural bioactive compounds. In particular, cinnamon *(Cinnamomun verum* J. Presl; syn. *Cinnamomun zeylanicum* Blume) and thyme (*Thymus vulgaris* L.) have been used by humans since ancient times due to their pesticidal, antimicrobial, antioxidant, and medicinal properties [1,2,3,4,5]. Essential oils (EOs) are among the most valuable fractions obtained from these aromatic species, comprising complex mixtures of secondary plant metabolites, including terpenoids and aromatic compounds.

The methoxyphenol eugenol is the major constituent of the *C. verum* essential oil [6]. The bioactivities exerted by essential oils containing eugenol are generally attributed to this compound [7,8]. Organic extracts of *C. verum*, of which aromatic compounds and carboxylic acids such as cinnamic and benzoic acid are reported as major compounds, also exhibit numerous bioactivities [9]. On the other hand, the essential oil of *T. vulgaris* contains phenolic monoterpenes as major constituents—particularly, thymol and its structural isomer carvacrol.

**Figure 1:**
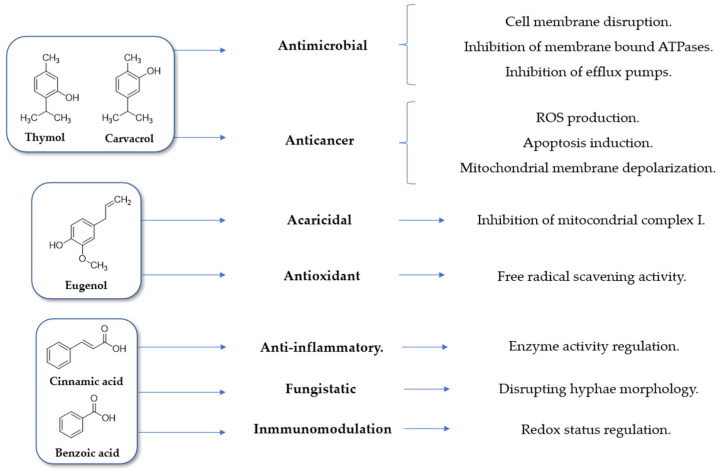
Main bioactivities and modes of action of the major constituents of the essential oils and organic extracts of *C. verum* and *T. vulgaris*.

These natural compounds have been extensively studied and may be responsible for the bioactivities of the essential oils containing them [10]. The modes of action of these natural bioactive compounds are under intense investigation, although some of them have been relatively well described. Figure 1 depicts some of the bioactivities exhibited by each compound described above and the modes of action by which they are exerted [11,12,13,14,15,16,17].

The structural motifs presented by these phytochemicals are responsible for their biological activities. The modification or hybridization of these structures could lead to the development of new naturally inspired compounds with potent biological activities, which could be used as a new generation of lead compounds. The design and synthesis of hybrid molecules based on bioactive phytochemicals would allow molecules to be obtained that are capable of interacting with different molecular targets simultaneously. Scientific evidence and cases of enormous commercial success support this notion [18,19]. Therefore, these hybrids could be used as multifunctional leads in novel biopesticide compositions that would minimize the appearance of resistance, or as the active ingredients of polypharmaceuticals, and have thus been proposed as a powerful tool to treat diseases that require complex therapeutic approaches [20,21].

In this work, we propose cost-effective synthesis and purification methods for six hybrid molecules based on the major constituents of *C. verum* and *T. vulgaris* EOs. We demonstrate, for the first time, their antifungal activity against two of most scientific/economic important phytopathogenic fungi, *Colletotrichum gloeosporioides* Penz. [22] and *Fusarium oxysporum* f. sp. *lactucae* [23], as well as one opportunistic human pathogen, *Aspergillus niger* P.E.L. To determine their antifungal activity, in vitro assays were carried out, covering two different modes of action. In addition, the cytotoxic activity was assessed in vivo by means of the brine shrimp lethality assay (BSLA), a well-established method for the preliminary screening of potential anticancer nature-based drugs. It has been proven that BSLA provides good correlation data with cytotoxic activity in some human solid tumors [24], such as 9KB cells (human nasopharyngeal carcinoma) and the 3PS (P388) cell line (leukemia in vivo) [25,26]. Although most of these hybrids have not yet been described in nature, we demonstrate here, for the first time, the process of obtaining these molecules by means of enzymatic catalysis.

The aim of this research was to provide a reliable method for the obtention of pure EO-based hybrid molecules which exert potent bioactivities, and to examine the possibility of obtaining them by natural means although they have not been reported in nature. These compounds thus have the potential to be used as novel active ingredients in strategic industries such as agriculture, aquaculture, and pharmaceuticals.

## 2. Materials and Methods

### 2.1. Chemicals and Reagents

Cinnamoyl chloride, benzoyl chloride, cinnamic acid, benzoic acid, eugenol, thymol, carvacrol, potassium hydroxide (KOH), anhydrous sodium sulfate (Na_2_SO_4_), ethyl acetate, n-hexane, and DMSO were purchased from Sigma-Aldrich (San Luis, MO, USA). The biocatalyst, Novozym^®^ 435, was purchased from STREM Chemicals Inc. (Newburyport, MA, USA).

### 2.2. Chemical Synthesis of the Hybrid Molecules

**Figure 2:**
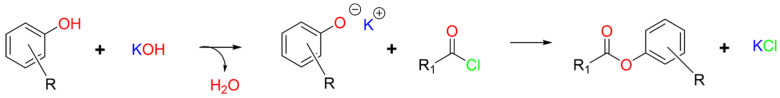
Chemical synthesis of the proposed hybrid molecules.

Chemical synthesis of the six hybrid molecules was achieved by means of bimolecular nucleophilic substitution. First, 50 mmol of the phenolic compound was stirred in a 10 mL KOH solution (5 mmol/mL) until neutralization. To the prior solution, 100 mmol of acyl chloride were slowly added. The mixture was continually stirred at room temperature (25 °C) for 2 h. The resulting mixture was submitted to liquid-liquid extraction, stirring it overnight with n-hexane and aqueous KOH saturated solution. The organic phase was recovered and the aqueous phase was discarded. The organic phase was dried over anhydrous sodium sulphate and filtered, and the solvent was evaporated under reduced pressure. Chemical characterization of products was carried out by means of GC-MS analysis. The structural features and purity of the obtained hybrid molecules were confirmed via ^1^H NMR analysis. Figure 2 shows the general reaction scheme for the chemical synthesis of the six hybrid molecules obtained.

### 2.3. Enzymatic Synthesis of the Hybrid Molecules

Enzymatic synthesis of the six hybrid molecules was carried out using Novozym^®^ 435 as a biocatalyst. Novozym^®^ 435 is *Candida antarctica* B lipase (CALB) immobilized on acrylic resin. First, 5 mmol of the phenolic compound were mixed with 5 mmol of carboxylic acid in a glass vial. Then, 0.1% (m/m) of Novozym^®^ 435 was added regarding the phenol. The reaction occurred over 72 h at 90 °C in the absence of solvent or agitation. The production of hybrid molecules was confirmed by means of GC-MS. The identification of products was made by comparing the retention time (t_R_) and mass spectra of the biosynthesized molecules with those obtained chemically.

### 2.4. GC-MS Analysis

The GC-MS analysis was carried out using an Agilent Model 7890 Series gas chromatograph (Agilent Technologies, Santa Clara, CA, USA), combined with an autosampler and an Agilent 7000 D GC/TQ mass-selective detector. Compounds were diluted in ethyl acetate at 1000 ppm and were separated on an HP-5MS UI 3 30 m long × 250 μm ID × 0.25 μm thick capillary column (Agilent Technologies, Santa Clara, CA, USA). The injection and ion source temperatures were 300 °C and 280 °C, respectively. Mass spectra were obtained at 70 eV in full-scan mode (40–500 *m*/*z*). The scan rate was 2.7 cycles/s. Compound identification was carried out via the comparison of the mass spectra obtained with fractionation patterns available in the NIST database (National Institute of Standards and Technology, Gaithersburg, MD, USA), and additionally via ^1^H NMR analysis.

The chromatographic conditions used were as follows: He as carrier gas at a flow rate of 1 mL/min; injector temperature = 300 °C, with a split ratio of 40:1; initial oven temperature = 70 °C (0 min), with a first ramp of 6 °C/min up to 250 °C (30 min), followed by a second ramp of 90 °C/min up to 290 °C, and a third stage where the temperature was maintained for 4 min. The total chromatography time was 34.4 min.

### 2.5. NMR Analysis

A Bruker Avance III 600 spectrometer, operating at a proton frequency of 600 MHz, equipped with a SampleJet 480-position thermostatised autosampler, and a QCI quadruple cryoprobe with magnetic field gradient, was used. Homonuclear (^1^H) NMR experiments were recorded. The acquisition and processing of the obtained spectra were performed using TOPSPIN software.

### 2.6. In Vitro Antifungal Activity Assay

Three phytopathogenic fungi: *Fusarium oxysporum f.* sp. *lactucae* J.C. Hubb. and Gerik, *Colletotrichum gloeosporioides* (Penzig) Penzig and Saccardo (CECT 21107), as well as an opportunistic human pathogen, *Aspergillus niger* P.E.L. van Tieghem 1867 (ATCC 9029), were used. Fungi were stored in the R&D department of Kimitec Group (Vícar, Almería, Spain) at −80 °C on glycerol (15%). Prior to the experiments, the microorganisms were activated at 28 °C on potato dextrose agar (PDA) medium for 7 days. The antifungal activity of the six hybrid molecules listed in Section 2.2 was evaluated for the inhibition of mycelial growth and spore germination.

#### 2.6.1. Mycelial Growth Inhibition

Hybrid molecules were dissolved on dimethyl sulfoxide (DMSO) at 50%, 25%, and 12.5% (*w*/*v*). Culture media were prepared mixing prior solutions at 1% (*v*/*v)* with PDA medium, achieving final concentrations of 0.5%, 0.25%, and 0.125%. Control treatments consisted of 1% (*v*/*v)* of DMSO with PDA medium. Media were autoclaved at 120 °C for 21 min and poured into Petri dishes. The inoculation was performed by placing an agar circle with mycelia from the cultures described in Section 2.6 in the center of each Petri dish. Next, the dishes were sealed with parafilm and incubated at 28 °C for 6 days. The diameter of the mycelium was measured on the sixth day. Five replicates per treatment were performed.

Mycelial growth inhibition was determined by measuring the mycelium diameter (cm) in the treatment samples, taking in consideration the growth observed in the control samples. The percentage of mycelial growth inhibition (MGI) was calculated according to the following formula [27]:

(1) $$\mathrm{MGI}=\frac{\mathrm{Dt}-\mathrm{Ds}}{\mathrm{Dt}}\times 100$$

where Dt is the diameter of the mycelium in the controls and Ds is the mycelium diameter in the treatment dishes.

#### 2.6.2. Spore Germination Inhibition

The effect of spore germination inhibition exhibited by the hybrid molecules was assessed by means of a previously reported slide assay [28] with some modifications. Briefly, a spore suspension was prepared by flooding 15 mL of potato dextrose broth (PDB) medium and scraping gently with a sterile Drigalski spatula on a 10-day culture of fungi grown in potato dextrose agar (PDA) medium at 28 °C. The spore suspension was filtered with sterile gauze. Then, the spore concentration was adjusted to 5 × 10^5^ spores/mL, using PDB medium and a Neubauer chamber for counting.

Hybrid molecules were dissolved in DMSO at 2.5%, 1.25%, and 0.625% (*w*/*v*). Then, 400 µL of the spore suspension and 100 µL of the hybrid molecule solution were homogenized via gentle agitation, reaching final concentrations of 0.5%, 0.25%, and 0.125%. The control group consisted of 400 µL of the spore suspension and 100 µL of DMSO. Then, 10 µL of the prior solution was placed in a glass slide and covered with a coverslip. All slides were incubated in a dark humidity chamber at 28 °C for 24 h. Using a light microscope (NIKON, Model Eclipse Ci-S, Tokyo, Japan) at 200× magnification, a total of 100 spores from each slide were counted, and the number of germinated spores was also determined. Germinated spores were those with a hyphal size at least as large as the spore diameter. The percentage of spore germination inhibition (SGI) was calculated using the following formula [29]:

(2) $$\mathrm{SGI}=\frac{\mathrm{Gc}-\mathrm{Gt}}{\mathrm{Gt}}\times 100\;$$

where Gc is the total number of germinated spores in the control group and Gt is the total number of germinated spores in the treatment group. Control and treatment samples were prepared in triplicate.

### 2.7. Brine Shrimp Lethality Bioassay

The cytotoxic effect of the six hybrid molecules was assessed by means of the brine shrimp lethality assay (BSLA) described by Meyer et al. [30]. The toxicities of hybrids were tested at 600, 300, 150, 100, 75, 50, 25, 10, and 1 µg/mL in 3 mL sea-water solutions in 1% DMSO (*v*/*v*). Sea water and DMSO were used as negative controls.

Briefly, *Artemia salina* cysts were purchased from IBERCAN-Spain, hatched, and maintained in laboratory for 48 h with continuous aeration at room temperature. In a 24-well plate, ten nauplii were disposed for each test in the presence of the prior solutions at the different concentrations. Three replicates were used for each concentration. After 24 h of exposure, the number of dead nauplii was counted and the percentage of mortality was calculated using the following formula [31]:

(3) $$\%\;\mathrm{mortality}=\frac{\mathrm{number}\;\mathrm{of}\;\mathrm{dead}\;\mathrm{nauplii}}{10\;\left(\mathrm{initial}\;\mathrm{number}\;\mathrm{of}\;\mathrm{live}\;\mathrm{nauplii}\right)\;}\times 100$$

Median lethal concentration (LC_50_) was determined for each hybrid molecule, using the regression line equation in the linear part of the graph by plotting the tested concentration against the death percentage. Survivors and dead nauplii were counted macroscopically by two independent counters.

### 2.8. Statistical Analysis

Statistical analysis was conducted using a one-way ANOVA and the results of biological activities were expressed as means plus standard deviations. Significant results were considered for *p*-values less than 0.05. In addition, Tukey’s test was carried out for the testing of multiple comparisons. STATGRAPHICS centurion Version 19.4.01 (64 bit) software was used for the statistical analysis.

## 3. Results

### 3.1. Chemical Synthesis of Hybrid Molecules Based on Major Constituents of Thymus vulgaris and Cinnamomun verum EOs

**Figure 3:**
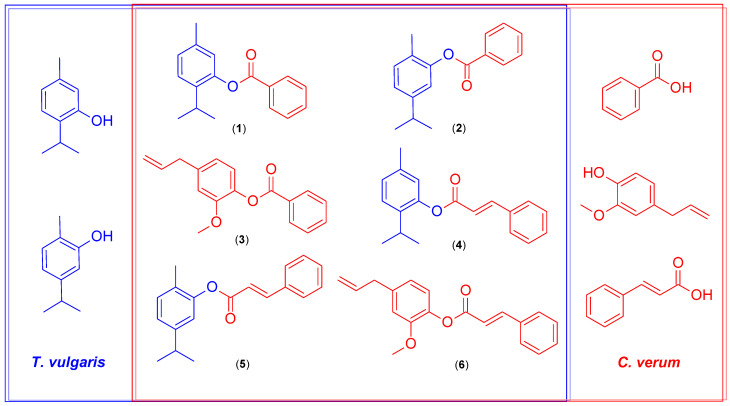
Chemical structures of hybrid molecules from the main constituents of *T. vulgaris* and *C. verum* EOs.

In total, six hybrid EO-based molecules were obtained via chemical synthesis. Figure 3 shows the structures of the six hybrids corresponding to esters of benzoic acid and cinnamic acid with the main constituents of *T. vulgaris* EO (thymol and carvacrol) and *C. verum* EO (eugenol).

The structures of the obtained molecules were established based on MS and ^1^H NMR analysis. Spectroscopic data were acquired in order to corroborate the structural features of the obtained hybrids. All structures have been previously reported in the literature [32,33,34,35,36,37]. Their yields and their chemical characterizations are presented below.

(**1**) 2-Isopropyl-5-methylphenyl benzoate [32]—Yellow liquid. Yield 70.93%; GC-MS (EI, 70 eV): *m*/*z* (%): 254 [M^+^] (38), 150 (34), 149 (26), 133 (17), 106 (11), 105 (100), 91 (28), 78 (24), 77 (24), 51 (21). ^1^H NMR (600 MHz, CDCl_3_) δ 8.24 (d, *J* = 7.4 Hz, 2H), 7.72–7.59 (m, 1H), 7.58–7.47 (m, 2H), 7.33–7.19 (m, 1H), 7.08 (dd, *J* = 8.0, 1.8 Hz, 1H), 6.97 (d, *J* = 0.9 Hz, 1H), 3.08 (hept, *J* = 6.9 Hz, 1H), 2.36 (s, 3H), 1.23 (d, *J* = 6.9 Hz, 6H).

(**2**) 5-Isopropyl-2-methylphenyl benzoate [32]—Orange liquid. Yield 75.04%; GC-MS (EI, 70 eV): *m*/*z* (%): 254 [M^+^] (15), 255 (33), 107 (17), 106 (20), 105 (100), 91 (45), 79 (16), 78 (38), 77 (38), 51 (34). ^1^H NMR (600 MHz, CDCl_3_) δ 8.24 (dd, *J* = 7.4, 1.3 Hz, 2H), 7.67–7.63 (m, 1H), 7.53 (dd, *J* = 8.3, 7.6 Hz, 2H), 7.23–7.17 (m, 1H), 7.10 (dd, *J* = 7.8, 1.8 Hz, 1H), 7.01 (d, *J* = 1.8 Hz, 1H), 2.92 (hept, *J* = 6.9 Hz, 1H), 2.22 (s, 3H), 1.26 (d, *J* = 6.9 Hz, 6H).

(**3**) 4-Allyl-2-methoxyphenyl benzoate [33,34]—Colorless crystals. Yield 71.15%; GC-MS (EI, 70 eV): *m*/*z* (%): 268 [M^+^] (9), 269 (2), 107 (1), 106 11), 105 (100), 103 (2), 91 (3), 78 (2), 77 (23), 51 (2). ^1^H NMR (600 MHz, CDCl_3_) δ 8.23 (d, *J* = 7.9, 2H), 7.63 (ddt, *J* = 8.8, 7.4, 1.3 Hz, 1H), 7.55–7.46 (m, 2H), 7.07 (d, *J* = 8.0 Hz, 1H), 6.87–6.75 (m, 2H), 6.03–6.95 (m, *J* = 1H), 5.15–5.11 (m, 2H), 3.81 (s, 3H), 3.42 (dt, *J* = 6.9, 1.6 Hz, 2H).

(**4**) 2-Isopropyl-5-methylphenyl cinnamate [35]—Yellow crystals. Yield 70.57%; GC-MS (EI, 70 eV): *m*/*z* (%): 280 [M^+^] (4), 149 (20), 135 (4), 132 (24), 131 (100), 104 (4), 103 (36), 102 (5), 91 (5), 77 (14). ^1^H NMR (600 MHz, CDCl_3_) δ 7.91 (d, *J* = 16.0 Hz, 1H), 7.65–7.60 (m, 2H), 7.52–7.41 (m, 3H), 7.33–7.18 (m, 1H), 7.13–7.06 (m, 1H), 6.97 (dd, *J* = 1.8, 0.9 Hz, 1H), 6.69 (d, *J* = 16.0 Hz, 1H), 2.92 (hept, *J* = 6.9 Hz, 1H), 2.20 (s, 3H), 1.27 (d, *J* = 7.0 Hz, 6H).

(**5**) 5-Isopropyl-2-methylphenyl cinnamate [35]—Pale brown crystals. Yield 78.12%; GC-MS (EI, 70 eV): *m*/*z* (%): 280 [M^+^] (2), 135 (3), 133 (2), 132 (12), 131 (100), 104 (2), 103 (19), 102 (3), 91 (3), 77 (8). ^1^H NMR (CDCl_3_, 600 MHz, δ, ppm): 7.91 (d, *J* = 16.0 Hz, 1H), 7.63 (m, 2H), 7.45 (m, 3H), 7.25 (d, *J* = 7.9 Hz, 1H), 7.20 (dd, *J* = 7.8, 1.8 Hz, 1H), 6.98 (d, *J* = 1.7 Hz, 1H), 6.70 (d, *J* = 16.0 Hz, 1H), 3.06 (hept, *J* = 5.9 Hz, 1H), 2.35 (s, 3H), 1.24 (d, *J* = 5.9 Hz, 6H).

(**6**) 4-Allyl-2-methoxyphenyl cinnamate [36,37]—Colorless crystals. Yield 75.44%; GC-MS (EI, 70 eV): *m*/*z* (%): 294 [M^+^] (3), 250 (2), 164 (3), 132 (14), 131 (100), 104 (3), 103 (24), 102 (3), 91 (3), 77 (9). ^1^H NMR (600 MHz, CDCl_3_) δ 7.88 (d, *J* = 16.0 Hz, 1H), 7.66–7.52 (m, 2H), 7.44–7.38 (m, 3H), 7.04 (d, *J* = 8.0 Hz, 1H), 6.88–6.78 (m, 2H), 6.68 (d, *J* = 16.0 Hz, 1H), 5.99 (ddt, *J* = 16.8, 10.0, 6.7 Hz, 1H), 5.20–5.02 (m, 2H), 3.84 (s, 3H), 3.41 (dt, *J* = 6.7, 1.5 Hz, 2H).

Except for (**3**), which was recently tentatively detected (but never isolated or quantified) as a minor compound in the non-polar fraction of the aerial part of *Vincetoxicum funebre* [38], the other five hybrids have never been reported in nature until now. However, in Section 3.2 we demonstrate the possibility of obtaining these compounds by natural means such as enzymatic catalysis.

**Figure 4:**
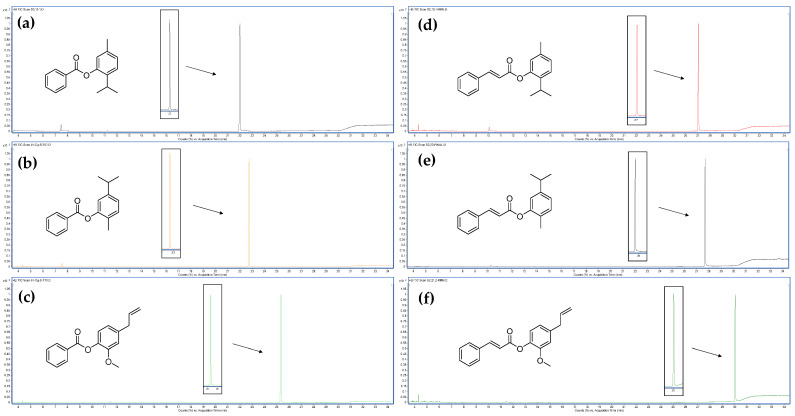
Chromatograms obtained via GC-MS of the chemical reaction products. (**a**) Chromatogram of 2-Isopropyl-5-methylphenyl benzoate (**1**); (**b**) chromatogram of 5-Isopropyl-2-methylphenyl benzoate (**2**); (**c**) chromatogram of 4-Allyl-2-methoxyphenyl benzoate (**3**); (**d**) chromatogram of 2-Isopropyl-5-methylphenyl cinnamate (**4**); (**e**) chromatogram of 5-Isopropyl-2-methylphenyl cinnamate (**5**); (**f**) chromatogram of 4-Allyl-2-methoxyphenyl cinnamate (**6**).

Following the described chemical synthesis method, six hybrids of ≥95% purity were obtained with high yields (≥70%) based on the spectroscopic analysis. All secondary products were obtained by following the proposed synthesis and purification method. Figure 4 shows the chromatograms acquired via GC-MS of the obtained compounds and the structures of each hybrid.

These results reveal that the proposed method of chemical synthesis could be reliable for obtaining further hybrid molecules based on the use of EOs in large quantities, specifically, via the esterification of different phenolic compounds with carboxylic acids, achieving good yields of high-purity products in two hours and in the absence of heat or hazardous solvents.

### 3.2. Enzymatic Synthesis of Hybrid Molecules Based on Major Constituents of Thymus vulgaris and Cinnamomun verum EOs

**Figure 5:**
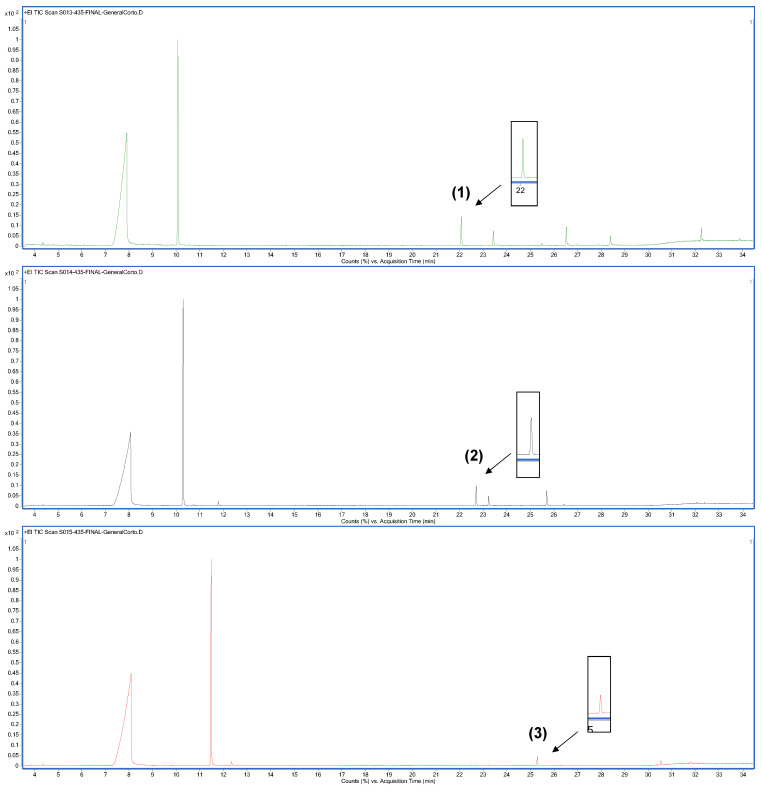
Chromatograms obtained via GC-MS of the enzymatic reaction products, obtaining (**1**), (**2**) and (**3**).

Although most of these hybrids were previously synthetized chemically, we have reported their obtention by means of enzymatic catalysis for the first time in absence of solvents. Figure 5 shows the chromatograms obtained via GC-MS of the enzymatic reactions when benzoic acid was used as the carboxylic acid and thymol, carvacrol, and eugenol were used as phenol compounds, obtaining hybrids (**1**), (**2**), and (**3**), respectively.

**Figure 6:**
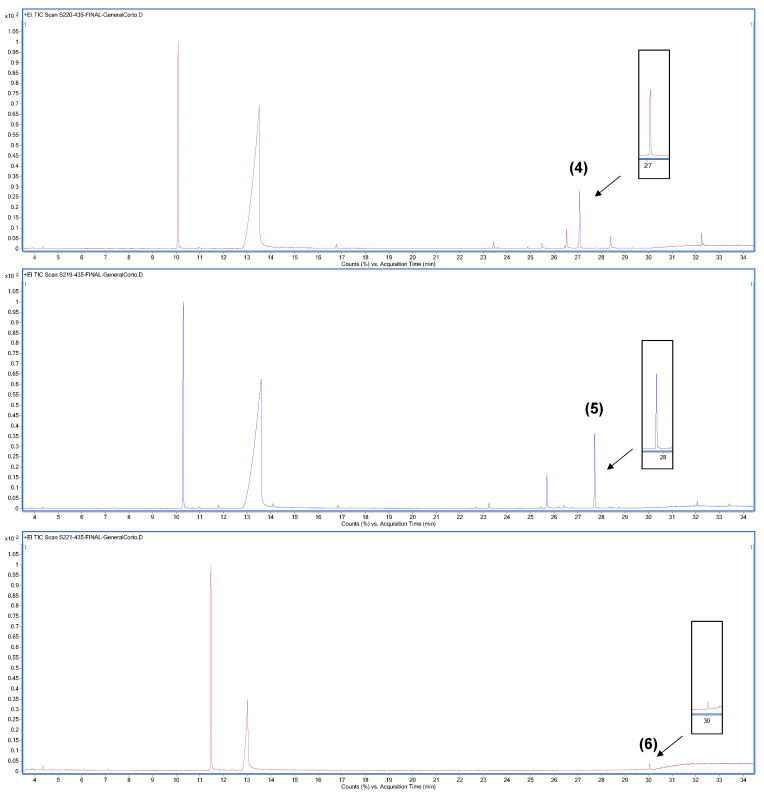
Chromatograms obtained via GC-MS of the enzymatic reaction products, obtaining (**4**), (**5**) and (**6**).

Figure 6 shows the chromatograms obtained for the enzymatic reaction products using cinnamic acid as the carboxylic acid. Thymol, carvacrol, and eugenol were the phenol compounds used to obtain hybrids (**4**), (**5**), and (**6**), respectively.

The peaks which correspond to target compounds have been pointed out with their numbers in the chromatograms. In all cases, the retention times and MS fractionation patterns coincided with those of the chemically obtained hybrids, allowing us to ensure that the chemically and enzymatically obtained compounds were the same.

Enzymatic reactions carried out under the proposed conditions produced qualitatively low product yields. Substrates, either carboxylic acid or phenols, were the major compounds at the end of the reaction, and several non-identified secondary products were obtained.

In view of these results, CALB lipase seems to be less specific to methoxyphenols such as eugenol and to have greater affinity for other phenylpropanoids such as cinnamic acid. The highest conversions of reactants to products were observed in reactions (**4**) and (**5**), when thymol, carvacrol, and cinnamic acid were used as substrates.

The possibility for the biotechnological production of these bio-based hybrid molecules was thus demonstrated, despite the fact that most of them have not previously been reported in nature. However, the method used in this study required subsequent purification processes in order to obtain large quantities of pure hybrid compounds based on the constituents of *T. vulgaris* and *C. verum* EOs. Temperature, the stoichiometric ratio of the substrate, and enzyme quantities could be also key variables in the optimization of the process.

The methodology and results described here for obtaining EO-based hybrids by means of enzymatic catalysis are in the process of being protected by a patent [39].

### 3.3. Biological Activity

The biological activity, in terms of antifungal activity against two modes of action, and the cytotoxic effects of the obtained EO-based hybrid molecules were assessed.

#### 3.3.1. Mycelial Growth Inhibition

Mycelial growth inhibition was evaluated by the so-called “poisoned food method” [40]. A mycelial disc of actively growing fungi was placed in the center of a Petri dish with PDA medium and the target hybrid molecule (DMSO in controls) at different concentrations. The radial growth of the mycelium was measured after 6 days of incubation and the percentage of mycelial growth inhibition (MGI) was calculated using the formula presented in Section 2.6.1. The efficacy of each hybrid molecule was expressed in terms of MGI against all fungi tested, as summarized in Table 1.

**Figure 7:**
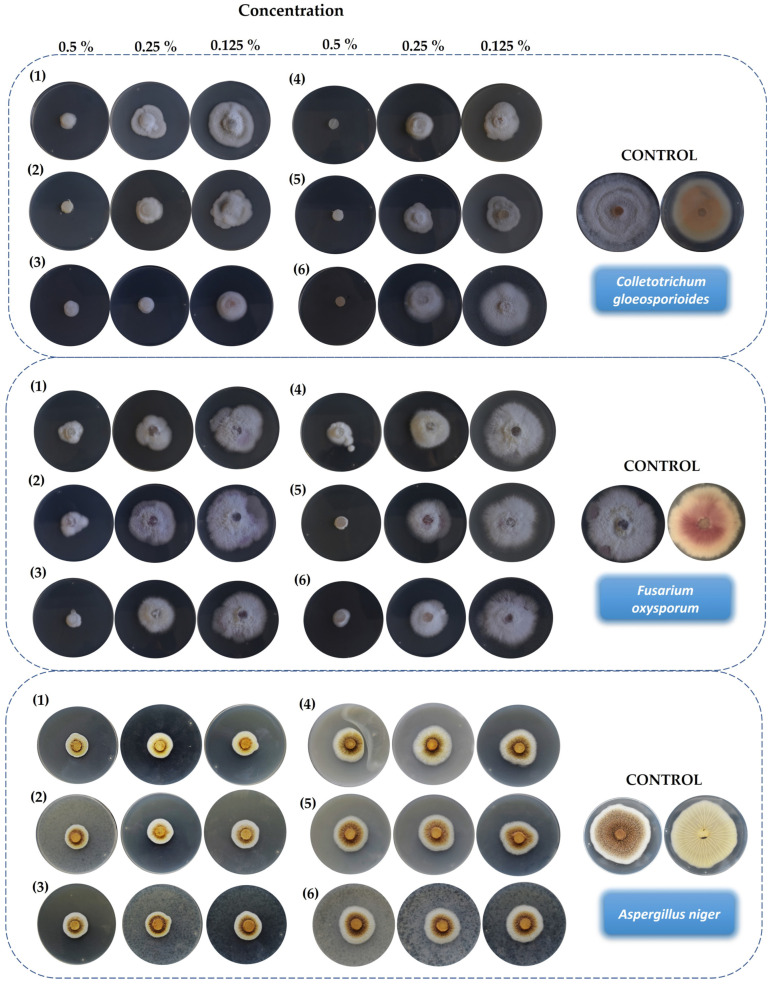
*Colletotrichum gloeosporioides, Fusarium oxysporum*, and *Aspergillus niger* mycelial growth inhibition effects exerted by hybrids: (**1**) 2-Isopropyl-5-methylphenyl benzoate, (**2**) 5-Isopropyl-2-methylphenyl benzoate, (**3**) 4-Allyl-2-methoxyphenyl benzoate, (**4**) 2-Isopropyl-5-methylphenyl cinnamate, (**5**) 5-Isopropyl-2-methylphenyl cinnamate, (**6**) 4-Allyl-2-methoxyphenyl cinnamate. Only one replicate per treatment and concentration is shown. Obverse and reverse of control treatment are shown.

The mycelial growth inhibition (MGI) effects against three fungi exerted by each hybrid molecule at three different concentrations are shown in Figure 7. In the case of *C. gloeosporioides*, all candidates showed strong antifungal activity at all concentrations tested. The use of hybrids (**4**) and (**6**) at 0.5% completely prevented the growth of fungi.

In comparison with *C. gloeosporioides*, hybrid molecules exerted lower antifungal efficacy against *F. oxyposrum*. At the highest concentration assayed, MGI was potent, but this effect nearly disappeared when hybrids were tested at 0.125%. Furthermore, cinnamic esters seemed to be more effective in inhibiting the mycelial growth of *F. oxysporum*.

In the case of *Aspergillus niger*, all hybrids exerted medium to low bioactivity. At 0.125%, (**4**) nearly lost its activity completely, but it became clear which hybrids from cinnamic acid exerted higher antifungal effects. It should be noted that the tested molecules did not exhibit dose-dependent response behavior.

In order to demonstrate the antifungal effects of the EO hybrid molecules, in Figure 7 we provide images of one replicate of each fungus at each concentration tested. Statistically significant differences between treatments were observed amongst the various concentrations assayed.

Although *A. niger* can also be responsible for plant diseases, is not a strictly phytopathogen. Consequently, these results indicated which of the hybrid phytochemical-based molecules tested, were able to exert potent mycelium inhibitory growth effects on specific plant pathogens but lesser effects on opportunistic human fungi.

#### 3.3.2. Spore Germination Inhibition

To obtain solid understanding of the compounds’ antifungal activity, we determined the spore germination inhibition (SGI) as a secondary mode of action, following the method described in Section 2.6.2. The results of the SGI analysis for each hybrid at three different concentrations against the selected fungi are shown in Table 2.

The SGI results obtained for the EO hybrid molecules can be qualitative extrapolated to describe their MGI bioactivities. All candidates exerted higher efficacy against *C. gloeosporioides* than against *F. oxyposrum*, and with regard to *A. niger* most of them were completely ineffective in inhibiting spore germination.

In general terms, all the concentrations tested exerted medium to low bioactivities. Once again, the obtained phytochemical hybrid molecules were relatively good spore-inhibitory ingredients for controlling phytopathogenic fungi but not for human diseases caused by these microorganisms.

**Figure 8:**
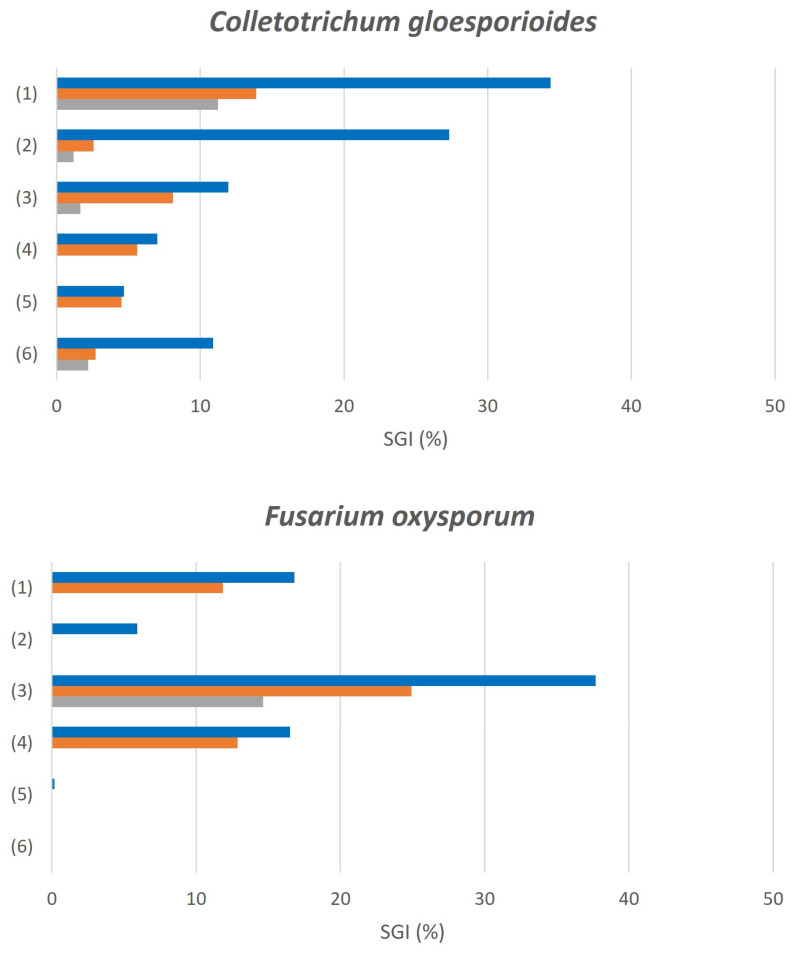
Spore germination inhibition effects exerted by hybrids against *Colletotrichum gloeosporioides* and *Fusarium oxysporum* at three different concentrations. Blue: 0.5% (*w*/*v*), Orange: 0.25% (*w*/*v*), Grey: 0.125% (*w*/*v*). (**1**) 2-Isopropyl-5-methylphenyl benzoate, (**2**) 5-Isopropyl-2-methylphenyl benzoate, (**3**) 4-Allyl-2-methoxyphenyl benzoate, (**4**) 2-Isopropyl-5-methylphenyl cinnamate, (**5**) 5-Isopropyl-2-methylphenyl cinnamate, (**6**) 4-Allyl-2-methoxyphenyl cinnamate.

Figure 8 contains a graphical depiction of the SGI results obtained for the hybrids to enable easier interpretation. As shown, for *C. gloeosporioides*, all hybrids displayed medium efficacy at 0.5% and the benzoic esters performed the best. For *F. oxysporum*, all hybrids exhibited medium to low activity at higher tested concentrations, with (**1**), (**3**), and (**4**) being the best candidates. In the case of *A. niger*, the SGI results are not shown in the graph because only (**1**) and (**2**) exerted low efficacies, and the other hybrids appeared to be completely ineffective in inhibiting spore germination. Most hybrids did not present dose-dependent response behavior in the SGI assays conducted in this study.

#### 3.3.3. Cytotoxic Activity

BSLA is a robust bioassay that is widely used in pharmacological studies for naturally occurring and natural-related chemicals, which is capable of identifying cytotoxic and bioactive compounds. Table 3 presents the cytotoxic activity in terms of the medium lethal concentrations for brine shrimp nauplii (LC_50_) of the six hybrid molecules examined in this study. Experiments were conducted for nine different concentrations—600, 300, 150, 100, 75, 50, 25, 10, and 1 µg/mL solutions in DMSO (1%).

According to the Clarkson’s toxicity criterion [41], plant extracts and natural compounds can be considered non-toxic when treatments have LC_50_ values above 1000 µg/mL, whereas LC_50_ between 1000–500 µg/mL are lowly toxic and treatments with LC_50_ 100–500 µg/mL and 100–0 µg/mL are moderately and highly toxic, respectively.

In view of these results, a clear relationship exist between the chemical structure and the mortality of *A. salina* nauplii. The eugenol-derived hybrids, (**3**) and (**6**), exerted medium–low toxic activities and exhibited LC_50_ values ten times higher than thymol and carvacrol derivatives, regardless of whether eugenol was hybridized with benzoic acid or cinnamic acid. Furthermore, hybrids based on benzoic acid appeared to be slightly more active than cinnamic acid derivatives and thymol-based molecules were more active than carvacrol-related hybrids. In view of the above conclusions and supported by the experimental data, (**1**) was the hybrid molecule with the lowest LC_50_ and the highest bioactivity against *A. salina*.

The hybridization of the major compounds of *C. verum* seemed to result in new molecules which did not exert high cytotoxic effects. Moreover, when hybrids were obtained by means of a combination of phenylpropanoids from *C. verum* and phenols from *T. vulgaris* EO, high cytotoxic effects were noted.

## 4. Discussion

In a changing scenario, in which the traditional active ingredients in agroindustry and pharmaceuticals are gradually being displaced by nature-based ingredients, basic and industrial research on novel lead compounds is necessary in order to comply with new legal and social requirements. Notably, in the European “Green Deal” strategy, 50% of conventional phytosanitary products will be prohibited by 2030 [42]. In the present study, we proposed to develop reliable chemical methods for the industrial scaling-up of six bioactive hybrid molecules based on major constituents of *Cinnamomun verum* and *Thymus vulgaris* EOs. Some recent works have demonstrated the possibility of obtaining, by other chemical methods, a few of these hybrid molecules, namely, 2-Isopropyl-5-methylphenyl benzoate (**1**), 4-Allyl-2-methoxyphenyl benzoate (**3**), and 2-Isopropyl-5-methylphenyl cinnamate (**4**), and their potential bioactivity as antileishmanial and insecticidal compounds has been tested [43,44]. For the first time, we have demonstrated further biological activities in the form of antifungal effects against two different modes of action, and their cytotoxicity results allowed us to propose industry-targeted applications for EO-based hybrid molecules. In addition, a distinguishing feature of the present research work is that it is the first demonstration of the obtention of these EO-based hybrid molecules by means of enzymatic catalysis, even though most of them have not been reported in nature.

To assess their antifungal activity, in vitro experiments were conducted using two different modes of action against the two most important phytopathogenic fungi and an opportunistic human pathogen. The inhibitory effects on the mycelial growth of *Colletotrichum gloeosporioides* and *Fusarium oxysporum* for 4-Allyl-2-methoxyphenyl benzoate (**4**) and 4-Allyl-2-methoxyphenyl cinnamate (**6**) were potent, and we determined that 0.5% was the concentration that inhibited 100% of mycelial growth. Regarding the inhibition of spore germination activities, benzoic acid derivatives exhibited the highest efficacies against *C. gloeosporioides* and *F. oxysporum*, but all of the compounds exerted medium to low efficacies. The obtained results indicated that these could be lead compounds in novel biopesticide formulations applied in field curative applications. Moreover, the next step to validate the antifungal activity of the selected hybrids could involve well-established in vivo pot or greenhouse assays against phytopathogenic fungi. In the other hand, the efficacy of the two modes of action against *Aspergillus niger* was quite low. Testing higher concentrations or searching for other phytochemical hybrids could provide ingredients for the treatment of opportunistic fungal human diseases. Even though the antifungal activities of all the starting products were well established [45,46,47,48], the aim of this study was to expand the chemical space, uncovering novel EO-based derivatives and determining their modes of action, as well as proposing new biotechnological means of obtaining them.

To examine the potential bioactivity of these EO-based molecules, the cytotoxicity was assessed by means of the brine shrimp lethality assay, a well-established method to preliminarily test the bioactivity of plant extracts and nature-related pure compounds. This easy, fast, accurate, and cost-effective bioassay is widely used for pre-screening anticancer compounds and extracts from natural sources [3,26,49]. The indicator of cytotoxicity (potential anticancer effects) is an LC_50_ value less than 1000 µg/mL. The EO-based hybrid molecules developed here exhibited a strong correlation between their toxicity against *A. salina* and their structural features. In all cases, they demonstrated potential as anticancer drugs, with toxicities varying between medium (LC_50_ ≈ 500 µg/mL) and high (LC_50_ ˂ 100 µg/mL). Eugenol-derived molecules were ten-fold less toxic than thymol or carvacrol hybrids, and benzoic derivatives were slightly more active. It is worth noting that the published data indicate that thymol’s activity against *A. salina* nauplii was LC_50_ = 514 µg/mL [30], ten times higher than that of 2-Isopropyl-5-methylphenyl benzoate (**1**) and 2-Isopropyl-5-methylphenyl cinnamate (**4**). For validation of the data obtained in the experiments performed here, the most effective hybrids, which correspond to the hybridization of phenolic monoterpenes from *T. vulgaris* and phenylpropanoids from *C. verum*, must be tested against target tumoral cell lines. These results could also imply toxicity to aquatic life; therefore, if applied in agriculture, well-established tests [50] to ensure ecotoxicological safety should be carried out. Alternatively, hybrids could be used which have been observed to exert high antifungal activities and low cytotoxicities, such as as 4-Allyl-2-methoxyphenyl benzoate (**3**).

In the present work, we have also amply demonstrated reliable, cost-effective, and industrially scalable chemical synthesis methods for EO-based hybrids. The high purity of the obtained products was demonstrated via H^1^ NMR, and the lowest yield achieved was higher than 70%. On the other hand, and although in this study we have presented the first demonstration of the enzymatic production of these hybrids, the proposed method demonstrates that they could be obtained by natural means, and thus could have the potential for industrial scalability. Further investigations could be accomplished regarding the enzymatic catalysis process. Variations in substrate ratios, temperatures, and reaction times, as well as the use of different enzymes and cost-effective purification processes, could be investigated in order to achieve higher conversion of the substrates to products or to avoid the obtention of secondary artefacts.

Specifically, the compound 2-Isopropyl-5-methylphenyl benzoate (**1**) was most active as an antifungal compound and as a potential anticancer drug, and was also one of the most reliable hybrids obtained via chemical and enzymatic methods. In conclusion, in the present work, we have proposed industrially-applicable chemical and enzymatic methods for obtaining hybrid molecules based on EO constituents. We have demonstrated which of these hybrids could represent novel active ingredients in agricultural biofungicide products and, potentially, in EO-based anticancer drugs. Furthermore, we revealed and discussed some structure–activity relationships, thus enabling the rational design of new EO-based hybrid molecules which could meet the demands of industry and society.

## Figures and Tables

**Table 1:** Inhibition values of hybrid molecules at different concentrations in regard to the mycelial growth of *C. gloeosporioides*, *F. oxysporum*, and *A. niger* at 6 days.

Hybrid	Mycelial Growth Inhibition (MGI)								
	*C. gloeosporioides*			*F. oxysporum*			*A. niger*		
	Concentration (*w*/*v*)			Concentration (*w*/*v*)			Concentration (*w*/*v*)		
	0.5%	0.25%	0.125%	0.5%	0.25%	0.125%	0.5%	0.25%	0.125%
(**1**)	94.76 ± 0.14 *	76.83 ± 0.12 *	65.56 ± 0.13 *	75.04 ± 0.14 *	63.20 ± 0.11 *	48.23 ± 0.07 *	65.13 ± 0.02 *	62.66 ± 0.05 *	57.19 ± 0.08 *
(**2**)	94.74 ± 0.06 *	86.77 ± 0.04 *	76.22 ± 0.09 *	69.74 ± 0.06 *	54.96 ± 0.08 *	29.48 ± 0.15 *	60.11 ± 0.02 *	58.97 ± 0.04 *	51.10 ± 0. 07 *
(**3**)	91.22 ± 0.08 *	88.24 ± 0.11 *	78.33 ± 0.10 *	83.09 ± 0.02 *	60.07 ± 0.10 *	51.48 ± 0.06 *	59.56 ± 0.03 *	57.27 ± 0.07 *	50.61 ± 0.03 *
(**4**)	100 ± 0.00 *	83.61 ± 0.04 *	73.82 ± 0.09 *	70.07 ± 0.18 *	61.62 ± 0.09 *	32.87 ± 0.12 *	18.34 ± 0.05 *	12.28 ± 0.08 *	3.31 ± 0.07 *
(**5**)	96.41 ± 0.03 *	80.67 ± 0.18 *	70.07 ± 0.09 *	86.82 ± 0.10 *	51.63 ± 0.07 *	30.81 ± 0.04 *	28.37 ± 0.06 *	23.10 ± 0.02 *	20.34 ± 0.05 *
(**6**)	100 ± 0.00 *	70.51 ± 0.04 *	64.23 ± 0.18 *	82.77 ± 0.08 *	64.23 ± 0.17 *	20.56 ± 0.05 *	27.92 ± 0.08 *	12.95 ± 0.11 *	11.19 ± 0.03 *

Values are presented as mean ± SD. * significantly different at *p* ˂ 0.05, as determined via the one-way ANOVA Tukey test.

**Table 2:** Inhibition of hybrid molecules at different concentrations on the spore germination of *C. gloeosporioides*, *F. oxysporum*, and *A. niger* at 24 h.

Hybrid	Spore Germination Inhibition (SGI)								
	*C. gloeosporioides*			*F. oxysporum*			*A. niger*		
	Concentration (*w*/*v*)			Concentration (*w*/*v*)			Concentration (*w*/*v*)		
	0.5%	0.25%	0.125%	0.5%	0.25%	0.125%	0.5%	0.25%	0.125%
(**1**)	34.40 ± 2.98 *	13.89 ± 1.84 *	11.24 ± 2.29	16.82 ± 0.25 *	11.84 ± 2.57	0.00 ± 0.00	13.68 ± 2.54 *	10.24± 1.87	0.00 ± 0.00
(**2**)	27.32 ± 1.86 *	2.57 ± 0.05	1.19 ± 0.76	5.92 ± 1.12	0.00 ± 0.00	0.00 ± 0.00	23.62 ± 3.05 *	10.91 ± 3.04	6.49 ± 1.12
(**3**)	11.94 ± 0.43 *	8.09 ± 0.30	1.64 ± 0.26	37.69 ± 6.3 *	24.92 ± 3.5 *	14.64 ± 0.75 *	0.00 ± 0.00	0.00 ± 0.00	0.00 ± 0.00
(**4**)	7.00 ± 1.89	5.60 ± 1.42	0.00 ± 0.00	16.52 ± 4.75 *	12.86 ± 1.23 *	0.00 ± 0.00	0.00 ± 0.00	0.00 ± 0.00	0.00 ± 0.00
(**5**)	4.68 ± 1.49	4.50 ± 0.93	0.00 ± 0.00	0.16 ± 0.11	0.00 ± 0.00	0.00 ± 0.00	0.00 ± 0.00	0.00 ± 0.00	0.00 ± 0.00
(**6**)	10.90 ± 5.3	2.72 ± 0.71	2.20 ± 0.87	0.00 ± 0.00	0.00 ± 0.00	0.00 ± 0.00	0.00 ± 0.00	0.00 ± 0.00	0.00 ± 0.00

Values are presented as mean ± SD. * significantly different at *p* ˂ 0.05 by means of the one-way ANOVA Tukey test.

**Table 3:** Percentage mortality of *Artemia salina* nauplii after 24 h treated with different concentrations of EO-based hybrid molecules and LC_50_ values calculated for each hybrid.

Hybrid	Concentrations (µg/mL)	% Mortality	LC_50_ (µg/mL)
(**1**)	600	100 ± 0.00 *	50.39
	300	100 ± 0.00 *	
	150	96.67 ± 5.77 *	
	100	76.67 ± 15.27 *	
	75	70.00 ± 10.00 *	
	50	46.67 ± 15.27 *	
	25	43.33 ± 10.00 *	
	10	33.33 ± 5.77 *	
	1	13.33 ± 5.77 *	
(**2**)	600	96.67 ± 5.77 *	68.22
	300	96.67 ± 5.77 *	
	150	83.33 ± 15.27 *	
	100	63.33 ± 15.27 *	
	75	60.00 ± 10.00 *	
	50	50.22 ± 10.00 *	
	25	30.68 ± 10.00 *	
	10	22.34 ± 0.00 *	
	1	10.12 ± 10.00 *	
(**3**)	600	66.67 ± 5.77 *	418.56
	300	26.67 ± 5.77 *	
	150	16.98 ± 5.77 *	
	100	16.42 ± 5.77 *	
	75	15.38 ± 0.00 *	
	50	12.11 ± 5.77 *	
	25	10.8 ± 0.00 *	
	10	8.7 ± 0.00 *	
	1	5.6 ± 0.00 *	
(**4**)	600	100 ± 0.00 *	64.41
	300	93.30 ± 5.77 *	
	150	86.67 ± 5.77 *	
	100	63.33 ± 5.77 *	
	75	60.00 ± 5.77 *	
	50	33.33 ± 5.77 *	
	25	36.67 ± 11.33 *	
	10	33.33 ± 5.77 *	
	1	20.00 ± 0.00 *	
(**5**)	600	80.00 ± 17.32 *	71.95
	300	70.00 ± 10.00 *	
	150	70.00 ± 10.00 *	
	100	63.33 ± 15.27 *	
	75	54.85 ± 5.77 *	
	50	40.00 ± 17.32 *	
	25	33.33 ± 5.77 *	
	10	36.67 ± 5.77 *	
	1	23.33 ± 0.00 *	
(**6**)	600	66.67 ± 15.27 *	465.87
	300	33.33 ± 5.77 *	
	150	30.00 ± 10.00 *	
	100	30.00 ± 0.00 *	
	75	23.33 ± 5.77 *	
	50	21.48± 5.77 *	
	25	16.67 ± 0.00 *	
	10	16.00± 5.77 *	
	1	15.00 ± 0.00 *	

Values are presented as mean ± SD. * significantly different at *p* ˂ 0.05 level by one-way ANOVA Tukey test.

## Data Availability

Not applicable.
